# Practice area and work demands in nurses' aides: a cross-sectional study

**DOI:** 10.1186/1471-2458-6-97

**Published:** 2006-04-13

**Authors:** Willy Eriksen

**Affiliations:** 1 Division of Mental Health, Norwegian Institute of Public Health, Oslo, Norway; 2 Institute of General Practice and Community Medicine, University of Oslo, Oslo, Norway

## Abstract

**Background:**

Knowledge of how work demands vary between different practice areas could give us a better understanding of the factors that influence the working conditions in the health services, and could help identify specific work-related challenges and problems in the different practice areas. In turn, this may help politicians, and healthcare administrators and managers to develop healthy work units. The aim of this study was to find out how nurses' aides' perception of demands and control at work vary with the practice area in which the aides are working.

**Methods:**

In 1999, 12 000 nurses' aides were drawn randomly from the member list of the Norwegian Union of Health – and Social Workers, and were mailed a questionnaire. 7478 (62.3 %) filled in the questionnaire. The sample of the present study comprised the 6485 nurses' aides who were not on leave. Respondents working in one practice area were compared with respondents not working in this area (all together). Because of multiple comparisons, 0.01 was chosen as statistical significance level.

**Results:**

Total quantitative work demands were highest in somatic hospital departments, nursing homes, and community nurse units. Physical demands were highest in somatic hospital departments and nursing homes. Level of positive challenges was highest in hospital departments and community nurses units, and lowest in nursing homes and homes or apartment units for the aged. Exposure to role conflicts was most frequent in nursing homes, homes or apartment units for the aged, and community nurse units. Exposure to threats and violence was most frequent in psychiatric departments, nursing homes, and institutions for mentally handicapped. Control of work pace was highest in psychiatric departments and institutions for mentally handicapped, and was lowest in somatic hospital departments and nursing homes. Participation in decisions at work was highest in psychiatric departments and community nurse units, and was lowest in somatic hospital departments and nursing homes.

**Conclusion:**

The demands and control experienced by Norwegian nurses' aides at work vary strongly with the practice area. Preventive workplace interventions should be tailored each area.

## Background

Nursing personnel are engaged in a wide range of practical and intellectual tasks and frequent social encounters at work, and are exposed to physical, as well as social, emotional, and intellectual work demands. These work demands do not necessarily represent a problem to the nursing staff, but if the demands are high, conflicting, or threatening, if there are few positive challenges, or if the demands are associated with low control, the result may be health problems, dissatisfaction, and high rates of sickness absence and turnover [1-8].

Knowledge of how work demands vary between different practice areas could give us a better understanding of the factors that influence the working conditions in the health services, and could help identify specific work-related challenges and problems in the different practice areas. In turn, this may give politicians a broader basis for their priorities and decisions, and it may help health-care administrators and managers to develop healthy work units. During the last two decades, a number of studies have examined the relationship between practice area and work demands in nursing personnel [9-17]. However, most of these studies examined registered nurses (graduate nurses) or mixed nursing personnel [9-16], whereas very few studies focused on nurses' aides (assistant nurses) [17], a large occupational group in many countries, with work demands differing from those of registered nurses. Studies were conducted in different parts of the world, including the USA [10,16], China [15], the United Kingdom [11-13], and New Zealand [9], but there are very few reports from Scandinavia [17]. Most studies were based on relatively small convenience samples. In a recent literature review, McVicar [18] concluded that "more such comparative studies are required".

In the Norwegian health services, there are obvious differences between the sectors with respect to the roles they are meant to fulfil, the types of patients they serve, and the characteristics of the organisational structures. Hospitals in Norway have the responsibility for treating severely and acutely ill patients, and to give highly specialised treatment. In these complex work organisations, there is a wide spectrum of personnel, and, consequently, many kinds of interactions between professionals, and the organisational structure of the work units is often hierarchical. As most hospital patients in Norway are being admitted for immediate help, the patient turnover is high. In the somatic departments, many patients are bedridden, whereas in the psychiatric wards, a large proportion of the patients are psychotic, in some cases violent.

In the Norwegian health services outside hospitals, the situation is different. As these services do not provide prestigious and technologically advanced treatments, their status among professionals and in the general public in Norway is lower, a fact that could reduce the grants they receive and the magnitude of resources they have at their disposal. The organisational structure of these work units is less hierarchical than in hospitals; in some units there is almost a flat structure, with a large number of personnel on the ground level, and only a few registered (graduate) nurses in charge. The patient turnover is relatively low, and the emotional bonds between personnel and patients have time to grow stronger. In Norwegian nursing homes, many inhabitants are bedridden, and in need of comprehensive care. In Norwegian homes or apartment units for the aged, the inhabitants are usually not bedridden, but in need of frequent help and to be looked after several times a day. Community nurses in Norway provide care in peoples' private homes, and their clients are people of all ages in need of help with certain limited problems (e.g. emotional support, monitoring the use of medicines, care of wounds that will not grow). In Norwegian institutions for mentally handicapped, the clients are usually not bedridden, but some are self-destructive and violent. In outpatient clinics, such as public polyclinics (for consultations with specialists) and offices of private doctors (general practitioners or specialists), the patients' functional level is usually high.

It seems likely that these variations in work settings may influence the level of demands and control at work among the nursing personnel. But we do not know how.

The aim of the present study was to find out how the perception of demands and control at work among Norwegian nurses' aides vary with the practice area in which the aides are working.

## Methods

### Participants and data collection

Nursing personnel in Norway include two large occupational groups: registered nurses, with at least three years training after high school, and certified nurses' aides, with either one year training after junior high school or a course that is part of a high school program. In addition, a smaller group of uncertified nurses' aides have no formal training and often hold temporary jobs. The number of vocationally active nurses' aides (both certified and unlicensed personnel) was estimated as approximately 55 000 in 1999 (Norwegian Union of Health – and Social Workers, personal communication). About 50 000 of these were members of the Norwegian Union of Health – and Social Workers (the Union).

In October 1999, 12 000 nurses' aides were drawn randomly from the Union's list of members, and were mailed a comprehensive questionnaire. The objective was to study working conditions, life-style, and health complaints in nurses' aides. After one reminder, 7478 (62.3 %) consented to participate in the study and filled in the questionnaire. The list of members also included persons who had retired from working life because of age, disability, or other reasons, and contacts over telephone during the data collection gave the impression that many of these non-working individuals were not motivated for participating in the study. Hence, the true response rate of the vocationally active subjects was probably higher than the overall response rate. The sample of the present study comprised the 6485 nurses' aides who were vocationally active and not on leave because of illness or pregnancy.

### Measurements

The type of ward was recorded by asking: "What is your main workplace?" The checklist had 12 optional answers that were not mutually exclusive: somatic hospital department for adults; psychiatric department or hospital for adults; paediatric department; polyclinic; nursing home; old people's home; unit of apartments for old people; community nurse service; institution for drug abusers; institution or dwelling unit for mentally handicapped; child care; other workplace.

Exposure to heavy physical work was measured with three questions exploring the frequency of moving patients manually in the bed, frequency of lifting or supporting patients manually between bed and chair, and frequency of lifting, carrying, or pushing heavy objects, such as heavy furniture and equipment. Optional answers were 0, 1–4, 5–9, and 10 or more times per shift. The first two questions were translations of questions developed and found valid by British scientists [19].

Psychological and social work demands and control at work were measured with questions from the General Nordic Questionnaire for Psychological and Social factors at Work (QPSNordic) [20]. Responses were scored on Likert five-point frequency scales (from '(1) never or very seldom' to '(5) very often or always'). Quantitative work demands were assessed by four questions (work piles up, have to work overtime, have to work in rapid pace, have too much to do). Positive challenges were assessed by three questions (work is challenging in a positive way, see the work as meaningful, job requires that you acquire new knowledge and skills). Role conflicts were measured with three questions (have to do things that you feel should be done differently, are given assignments without adequate resources, receive incompatible requests from two or more people). Exposure to threats or violence was measured with one question. Control of work pace was measured with three questions (can set your own work pace, can decide when to take a break, can set your own working hours). Participation in important decisions was assessed by three questions (can choose which method to use for doing your work, can influence the amount of work, can influence decisions that are important for your work). The work factors that were assessed by several questions, were expressed as indices, calculated as the mean of the item scores. The internal consistency (Cronbach's alpha) of the indices was in the present study in the range of 0.68 to 0.88, except the index of control of work pace (0.57).

### Ethics

The research protocol was approved by the Committee for Medical Research Ethics. Informed written consent was given by the responders.

### Statistical analyses

As the level of demands and control at work was not normally distributed, Mann-Whitney rank sum tests and logistic regression analyses were used to explore differences between practice areas. In these analyses, respondents working in one practice area were compared with respondents not working in this area (all together). Some areas were represented by few respondents, and were not examined. Because of multiple comparisons, 0.01 was chosen as statistical significance level.

In the logistic regression analyses, the dependent variables were dichotomised, with "high score" defined as values higher than the median. Adjustments were made for age, gender, and marital status, with all covariates entered simultaneously.

## Results

### Characteristics of the sample

The demographic characteristics of the sample were as follows: 247 were men, and 6234 were women. 505 were younger than 30 years of age, 1318 were 30–39 years, 2612 were 40–49 years, 1764 were 50–59 years, and 284 were 60 years or older. 5228 were married or cohabiting, and 1244 were single.

The practice areas in which the respondents were working, and the relationship between practice areas and demographic characteristics, are presented in Table 1. The majority of the respondents were working in practice areas outside hospitals. The proportion of respondents older than 44 years was higher in hospital departments than in practice areas outside hospitals. The proportion of males was highest in psychiatric departments and institutions for mentally handicapped. The proportion of singles was higher in hospital departments than in practice areas outside hospitals. The perceived levels of demands and control at work are presented in Table 2.

### Practice area and demands and control

The perceived level of demands and control at work varied strongly between practice areas (Tables 3, 4, 5, 6). The associations that were found in the univariate analyses (Tables 3 and 4), were, in most cases, also seen in the multivariate logistic regression analyses, after adjustments for age, gender, and marital status (Tables 5 and 6). As shown in Tables 5 and 6, high quantitative work demands were more often reported in somatic hospital departments, nursing homes, and community nurse units than in other practice areas, and were less often reported in psychiatric departments, homes or apartment units for the aged, and institutions for mentally handicapped than in other practice areas. High physical demands were more often reported in nursing homes and somatic hospital departments, and were less often reported in psychiatric and paediatric departments, community nurse units, and institutions for mentally handicapped. High positive challenges were more often reported in hospital departments and community nurse units, and were less often reported in nursing homes and homes or apartment units for the aged. Frequent exposure to role conflicts was more often reported in nursing homes, homes or apartment units for the aged, and community nurse units, and was less often reported in hospital departments. Frequent exposure to threats and violence was more often reported in psychiatric departments, nursing homes, and institutions for mentally handicapped, and was less often reported in somatic departments, paediatric departments, and community nurse units. High control of work pace was more often reported in psychiatric departments and institutions for mentally handicapped, and was less often reported in somatic departments and nursing homes. High participation in decisions at work was more often reported in psychiatric departments and community nurse units, and was less often reported in somatic hospital departments and nursing homes.

## Discussion

In this survey of Norwegian nurses' aides, perceived demands and control at work varied strongly with the practice area in which the aides were working.

### Strengths and weaknesses of the study

The study was based on a large, randomly selected, nation-wide sample. The relative homogeneity of the participants in educational attainment and occupation served to enhance the internal validity of the study. The response rate was not optimal, though (62 %).

The instruments that were used to measure psychological demands and control at work have been found to have good construct and predictive validity as well as good internal consistency and test-retest reliability in a heterogeneous population from Denmark, Sweden, Finland, and Norway [20]. Studies of Norwegian nurses' aides, based on the sample that was used in the present study, have shown that these instruments may help predict sickness absence [3-5], smoking relapse in former smokers [21], and a range of other outcomes, including psychological distress, sleep problems, persistent fatigue, and intentions to quit [Eriksen W, unpublished results], although not all factors predict all outcomes.

The two questions used to assess the frequency of patient handling have been found to have good validity [19], and have been found to predict back pain in British nurses [6]. Studies of Norwegian nurses' aides, based on the sample that was used in the present study, have shown that the question about the frequency of positioning patients in bed may help predict low back pain [5], and the question about the frequency of handling of heavy objects may help predict both all-cause sickness absence [3] and sickness absence attributed to low back pain [5].

### Comparison with other studies

Whereas most other studies of the relationship between practice area and work demands in nursing personnel have examined registered nurses or mixed nursing personnel [9-16], the present study is one of very few studies that have focused on nurses' aides. Abrahamsen [17] also examined Norwegian nurses' aides, but her study was based on a smaller sample that was not randomly selected, and the measurements of work demands were less specific than the measurements in the present study. The study of Abrahamsen showed, in agreement with the present study, that the physical demands were highest in nursing homes and somatic hospital departments. On the other hand, Abrahamsen found that the psychological demands (unspecified) were highest in psychiatric wards and institutions for mentally handicapped. In the present study, exposure to threats and violence was found to be high in these two practice areas, but quantitative work demands and exposure to role conflicts were relatively low, and the level of control was high.

### Explanations of the associations between practice area and demands and control

High quantitative work demands (have to work in rapid pace, work piles up etc.) were seen in hospitals (somatic departments) as well as in practice areas outside hospitals (nursing homes and community nurse units). These work units are quite different from each other in patient turnover, organisational structure, and professional prestige. However, both somatic hospital departments and nursing homes serve patients with severe physical disorders, a fact that is likely to have a strong impact on the magnitude of the aides' work tasks. In community nurse units, it may be difficult to limit the number of patients under treatment, and the distance from one patient to another may be long. Hence, the number of personnel that is needed in these work units may easily be underestimated by those responsible for the personnel situation. High physical demands were seen in both nursing homes and somatic hospital departments. The reason is obviously the type of patients that are served in these work units.

The level of positive challenges was high in hospital departments and community nurse units, and was low in nursing homes and homes or apartment units for the aged. Patients in Norwegian hospitals represent all age-groups and are offered a large variation of treatments, and this may probably give more variation in the aides' tasks. In Norwegian community nurse units, the aides are working very independently out in the field, and they are often doing the same tasks as registered nurses. In nursing homes and homes or apartment units for the aged, where the inhabitants are old and often dying, the attitudes towards medical treatments may be less offensive, even pessimistic, and the personnel may easily become disappointed and end up with a fatalistic way of thinking ("whatever we do, the patients die").

The exposure to role conflicts at work was high in service sectors outside the hospitals (nursing homes, homes or apartment units for the aged, community nurse units), and was low in hospital departments. One explanation could be that the emotional bonds between personnel and patients are stronger outside hospitals; with strong emotional bonds to patients, the personnel may be more inclined to be affected when the resources to fulfil the assignments are not adequate, or when they receive incompatible requests from two or more patients. Another possible explanation may be that the organisational structure of the services outside hospitals is less hierarchical, and the management weaker. This increases the risk that informal leaders in the staff appear and put personnel in situations with conflicting loyalty and demands.

The frequent exposure to threats and violence in psychiatric wards, and to some extent also in nursing homes and institutions for mentally handicapped, is due to the types of patients in these practice areas. Psychotic patients as well as patients with mental handicaps or dementia may in some cases be violent.

The control that the aides have on the work pace seems to be low in somatic departments and nursing homes (where patients are affected by serious physical disorders), and seems to be high in psychiatric wards and institutions for mentally handicapped (where mental disorders represent the challenges). The urgency of basic, physiological functions, and the extent the different types of patients need help with these functions, may be the main cause of this variation in the control of work pace. Helping patients to the toilet, bringing bedpans, changing dirty sheets, and helping patients with the meals are examples of tasks that may be difficult to postpone – and tasks that nursing personnel in somatic hospital departments and nursing homes are often confronted with.

The participation in decisions at work was high in psychiatric departments and community nurse units, and low in somatic hospital departments and nursing homes. In psychiatric departments, the therapeutic meetings and milieus may perhaps level out the formal differences between various types of personnel, and may give nurses' aides a "hand on the wheel". In community nurse units, the aides are working independently out in the field, and will therefore have a strong influence on decisions at work. The low participation in somatic hospital departments may be a result of the hierarchical structure of these organisations in Norway. In Norwegian nursing homes, the organisational structure is not so hierarchical, but it is registered nurses, and not nurses' aides, who are in charge. Informal leaders and struggles for power in the nursing homes could perhaps also reduce the aides' participation in decisions. One should take into account that individual-level characteristics, such as personality factors, may have influenced both job preferences and the perception of work demands and control, and may, hence, also have contributed to the statistical associations between practice area and work factors.

### Implications

Workplace interventions are complex processes, which often require participation by the whole work organisation, and, in some cases, also require purchase of equipment or expertise. Such interventions are therefore expensive, time-consuming, and difficult to implement. As a consequence of this, focusing on changing only the most problematic factors in a workplace may be the most cost-beneficial procedure.

Exposure to threats and violence seems to be a major problem for nurses' aides who are working in psychiatric departments and institutions for mentally handicapped in Norway. The violence problem should, therefore, be given high priority in these sectors. Correct treatment of the patients is probably the most important measure to prevent violent incidents. Hence, training of the staff is essential. Adequate number of personnel on the shift is also important. Detailed plans for how to deal with violent patients should be made. Such plans, including rules for the number of personnel on shift, and descriptions of correct behaviour and ways to call for and give assistance, may not only prevent episodes of violence, but may also give the personnel a feeling of control, which has been shown to moderate the psychological impact of the violence [22]. Support from colleagues and superiors during and after incidents of violence is also important to reduce psychological distress among the personnel [23].

The main problems for nurses' aides who are working in Norwegian community nurse units seem to be that they have too much to do and are frequently exposed to role conflicts. More personnel is probably needed to solve these problems.

The main problems for nurses' aides who are working in Norwegian homes and apartment units for the aged seem to be lack of positive challenges and frequent exposure to role conflicts at work. Regular courses and theoretical training could help these aides to identify positive challenges in their work. Easier access to supervision, help, and equipment when the inhabitants (who are usually not bedridden) become temporarily disabled by intercurrent diseases, may also improve the work situation of these aides.

The spectrum of problems is somewhat wider in somatic hospital departments for adults in Norway. In these work units, the aides report high quantitative work demands and high physical demands, as well as little influence on the work situation. More personnel would reduce quantitative and physical demands, and would probably also increase the control of work pace. The needs may vary between the different types of wards (surgery, internal medicine, neurology, dermatology etc), and further research is needed to determine these variations.

The situation for nurses' aides in Norwegian nursing homes seems to be very problematic. According to the present study, quantitative demands and physical demands, as well as exposure to role conflicts and threats and violence at work are higher in this practice area than in other areas, whereas positive challenges and control at work are lower. As reported in an earlier study [24], also perceived social support at work seems to be low among nurses' aides in Norwegian nursing homes. Obviously, there is an urgent need of interventions in Norwegian nursing homes, and these interventions have to be broad-based and comprehensive. More personnel are needed, including nurses' aides, registered nurses, physiotherapists, and doctors. There is probably also a need of more equipment, including devices to support patient transfer procedures (hoists etc.), and the staff should be given more training in the use of this equipment. Organising work in another way, and increasing aides' opportunity to participate in important decisions at work should be considered. External consultants, such as organisational psychologists, may be hired to help understand the organisational challenges.

## Conclusion

The demands and control experienced by Norwegian nurses' aides at work vary strongly with the practice area in which the aides are working. These variations should be taken into account by politicians, administrators, and managers in order to develop healthy work units. Nursing homes need extra attention, as the working conditions in these work units are very problematic.

## Competing interests

The author(s) declare that they have no competing interests.

## Pre-publication history

The pre-publication history for this paper can be accessed here:



## Figures and Tables

**Table 1:** Age, gender, and marital status, by practice area.

Practice area		45 years or older	Male	Single
	N	Row %	Row %	Row %
Somatic hospital department	840	64.1 **	2.3	22.7 *
Psychiatric hospital department	466	58.2 **	13.7 **	21.5
Paediatric hospital department	115	59.1	0.9	23.5
Nursing home	2750	49.4 **	1.9 **	18.0
Home or apartment unit for the aged	640	50.5	3.1	17.5
Community nurse unit	1003	45.8 **	2.8	20.2
Institution for mentally handicapped	672	45.8 **	8.6 **	19.0
Other	392	53.8	4.8	17.1
				
Total sample	6485	51.6	3.8	19.2

N = The number of respondents working in each practice area; as the categories are not mutually exclusive, the sum of respondents in each area is higher than the total sample.

Row % = The proportion of respondents with the noted characteristic; for example, the proportion of male respondents in somatic hospital departments was 2.3 %.

* P < 0.01; ** P < 0.001.

The P-values refer to chi square tests, in which respondents working in the noted practice area are compared with respondents not working in this area (all together).

**Table 2:** Demands and control at work.

Factor	N	%	Mean	SD	Range
Quantitative work demands			2.89	0.76	1.00 – 5.00
Positioning patients in bed ^†^					
0	1132	18.3			
1–4	2702	43.6			
5–9	1526	24.6			
10 or more	834	13.5			
Supporting patients between bed and chair ^†^					
0	1049	17.1			
1–4	2752	44.8			
5–9	1579	25.7			
10 or more	763	12.4			
Handling heavy objects ^†^					
0	1758	29.1			
1–4	3311	54.9			
5–9	675	11.2			
10 or more	288	4.8			
Positive challenges			2.90	0.71	1.00 – 5.00
Role conflicts			2.38	0.81	1.00 – 5.00
Exposure to threats and violence					
Never or very seldom	3970	61.5			
Rather seldom	834	12.9			
Sometimes	1084	16.8			
Rather often	442	6.8			
Very often or always	127	2.0			
Control of work pace			2.36	0.80	1.00 – 5.00
Participation in important decisions			3.31	0.80	1.00 – 5.00

N = Number of respondents in each category. SD = Standard deviation. ^†^Times per shift. The variables for which the mean, standard deviation, and range are shown, are indices of several items, whereas the variables for which numbers and percentages are shown, have been measured with a single question.

**Table 3:** Demands and control at work, by practice area. Hospital departments. Results of Mann-Whitney rank sum tests.

Work factors	Somatic department (adults)	Psychiatric department (adults)	Paediatric department
Quantitative work demands	3758 (3153) **	2052 (3322) **	3266 (3230)
Positioning patients in bed	3322 (3064) **	1189 (3241) **	1943 (3117) **
Supporting patients between bed and chair	3033 (3078)	1200 (3210) **	1510 (3098) **
Handling heavy objects	3241 (2983) **	1936 (3099) **	2853 (3019)
Positive challenges	3611 (3172) **	3707 (3191) **	3878 (3217) **
Role conflicts	2998 (3269) **	2785 (3255) **	2541 (3233) **
Exposure to threats or violence	2387 (3354) **	4652 (3119) **	2124 (3249) **
Control of work pace	2752 (3301) **	3903 (3178) **	2895 (3236)
Participation in important decisions	2970 (3259) **	3689 (3186) **	3054 (3225)

The figures are mean ranks of participants working in the noted practice area, and, in paranthesis, mean ranks of the participants not working in this area. For example, the figures in the upper left corner, 3758 (3153), show that the level of quantitative work demands was higher in somatic departments (mean rank = 3758) than in other practice areas all together (mean rank = 3153). * P < 0.01; ** P < 0.001.

**Table 4:** Demands and control at work, by practice area. Practice areas outside hospital. Results of Mann-Whitney rank sum tests.

Work factors	Nursing home	Home or apartment unit for the aged	Community nurse unit	Institution for mentally handicapped
Quantitative work demands	3401 (3106) **	2989 (3257) *	3742 (3137) **	2377 (3329) **
Positioning patients in bed	3962 (2436) **	3102 (2097)	2977 (3120)	1726 (3248) **
Supporting patients between bed and chair	3837 (2488) **	3362 (3041) **	3027 (3080)	1969 (3194) **
Handling heavy objects	3320 (2790) **	3083 (3009)	3010 (3018)	2577 (3066) **
Positive challenges	2912 (3461) **	2934 (3261) **	3640 (3153) **	2971 (3258) **
Role conflicts	3373 (3109) **	3441 (3197) *	3619 (3148) **	3204 (3223)
Exposure to threats or violence	3362 (3131) **	3079 (3245)	2932 (3283) **	3720 (3172) **
Control of work pace	3017 (3387) **	3326 (3219)	3353 (3208)	3914 (3151) **
Participation in important decisions	3087 (3322) **	3127 (3232)	3480 (3175) **	3283 (3215)

The figures are mean ranks of participants working in the noted practice area, and, in paranthesis, mean ranks of the participants not working in this area. For example, the figures in the upper left corner, 3401 (3106), show that the level of quantitative work demands was higher in nursing homes (mean rank = 3401) than in other practice areas all together (mean rank = 3106). * P < 0.01; ** P < 0.001.

**Table 5:** The odds of high values (> median) of demands and control at work. Hospital departments. Results of 27 logistic regression analyses.

Work factors	Somatic department (adults)	Psychiatric department (adults)	Paediatric department
Quantitative work demands	1.64 **	0.27 **	0.82
Positioning patients in bed	1.27 *	0.06 **	0.13 **
Supporting patients between bed and chair	0.82	0.06 **	0.08 **
Handling heavy objects	1.30 *	0.19 **	0.82
Positive challenges	1.45 **	1.59 **	1.80 *
Role conflicts	0.79 *	0.63 **	0.52 *
Exposure to threats or violence	0.23 **	6.09 **	0.075 **
Control of work pace	0.59 **	1.90 **	0.71
Participation in important decisions	0.80 *	1.68 **	0.86

The figures are odds ratios, adjusted for age, gender, and marital status, and where respondents who were not working in the noted practice area have been used as reference category. For example, the figure in the upper left corner (1.64) shows that the odds of reporting a high value of quantitative work demands were 64 % higher in somatic departments than in other practice areas all together. * P < 0.01; ** P < 0.001.

**Table 6:** The odds of high values (> median) of demands and control at work. Practice areas outside hospital. Results of 36 logistic regression analyses.

Work factors	Nursing home	Home or apartment unit for the aged	Community nurse unit	Institution for mentally handicapped
Quantitative work demands	1.29 **	0.72 **	1.69 **	0.44 **
Positioning patients in bed	5.29 **	0.79	0.51 **	0.13 **
Supporting patients between bed and chair	4.37 **	1.17	0.61 **	0.25 **
Handling heavy objects	1.84 **	1.00	0.71 *	0.43 **
Positive challenges	0.57 **	0.68 **	1.57 **	0.84
Role conflicts	1.34 **	1.31 *	1.51 **	0.81
Exposure to threats or violence	1.39 **	0.84	0.71 **	1.64 **
Control of work pace	0.73 **	1.11	1.20	2.20 **
Participation in important decisions	0.80 **	0.88	1.30 **	1.01

The figures are odds ratios, adjusted for age, gender, and marital status, and where respondents who were not working in the noted practice area have been used as reference category. For example, the figure in the upper left corner (1.29) shows that the odds of reporting a high value of quantitative work demands were 29 % higher in nursing homes than in other practice areas all together. * P < 0.01; ** P < 0.001.
